# Update on Senecavirus Infection in Pigs

**DOI:** 10.3390/v9070170

**Published:** 2017-07-03

**Authors:** Raquel A. Leme, Alice F. Alfieri, Amauri A. Alfieri

**Affiliations:** 1 Laboratory of Animal Virology, Department of Veterinary Preventive Medicine, Universidade Estadual de Londrina, P.O. Box 10011, Paraná 86057-970, Brazil; raquelarrudaleme@gmail.com (R.A.L.); aalfieri@uel.br (A.F.A.); 2 Multi-User Animal Health Laboratory, Molecular Biology Unit, Department of Veterinary Preventive Medicine, Universidade Estadual de Londrina, P.O. Box 10011, Paraná 86057-970, Brazil

**Keywords:** swine, picornavirus, Seneca Valley virus, emergent disease, vesicular disease, neonatal mortality

## Abstract

*Senecavirus A* (SVA) is a positive-sense single-stranded RNA virus that belongs to the *Senecavirus* genus within the *Picornaviridae* family. The virus has been silently circulating in pig herds of the USA since 1988. However, cases of senecavirus-associated vesicular disease were reported in Canada in 2007 and in the USA in 2012. Since late 2014 and early 2015, an increasing number of senecavirus outbreaks have been reported in pigs in different producing categories, with this virus being detected in Brazil, China, and Thailand. Considering the novel available data on senecavirus infection and disease, 2015 may be a divisor in the epidemiology of the virus. Among the aspects that reinforce this hypothesis are the geographical distribution of the virus, the affected pig-producing categories, clinical signs associated with the infection, and disease severity. This review presents the current knowledge regarding the senecavirus infection and disease, especially in the last two years. Senecavirus epidemiology, pathogenic potential, host immunological response, diagnosis, and prophylaxis and control measures are addressed. Perspectives are focused on the need for complete evolutionary, epidemiological and pathogenic data and the capability for an immediate diagnosis of senecavirus infection. The health risks inherent in the swine industry cannot be neglected.

## 1. Introduction

A new virus named Seneca Valley virus was identified in 2002, and classified as the only representative strain of the *Senecavirus A* species in the new genus *Senecavirus*. Since then, until the end of 2014, little progress has been made regarding the virus features and infection in swine, including its epidemiology, transmission, pathogenesis, pathology, clinical signs, and diagnosis. The disease was considered exotic and limited in terms of geographic distribution. Senecavirus infection in swine has not been extensively studied by North American researchers or by worldwide research groups, even though it is a vesicular disease, likely due to the low frequency of infection, with the only reported cases being in North American countries.

In early 2015, different isolates of senecavirus were first reported in vesicular disease outbreaks outside of North America in Brazil, China, and Thailand. In the same year, the virus infection was associated with novel clinical manifestations in newborn pigs. Simultaneously, new cases occurred in the USA. These events were responsible for an increase in the number and the diversity of research approaches based on the senecavirus infection.

More knowledge has been accumulated and more unknown points have been clarified on the etiological agent and the virus infection, in approximately the last 24 months, than have been in all of the previous years. The scientific community was able to comply promptly with the demand for objective and clear answers to issues raised by pig producers and consultant veterinarians in private and public sectors who suddenly faced outbreaks of a completely unknown vesicular infectious disease. 

The currently available data were generated in a short timeframe, which is rarely seen in similar cases of viral diseases in animals without a public health impact. The goal of this review is to present the step-by-step evolution of senecavirus-based studies, particularly in the last two years, and to describe the features of the etiological agent and infection in pigs.

## 2. History, Classification, and Molecular Features

Seneca Valley virus was incidentally isolated in Gaithersburg, Maryland, USA in 2002 from the PER.C6 (transformed fetal retinoblast) cell line. The virus had evidently been introduced into the cell culture through the use of contaminated fetal bovine serum or porcine trypsin [1]. The virus was discovered in the laboratories of Neotropix, Inc., located near Seneca Creek State Park, Gaithersburg, MA, USA, which explains the name of the virus [2]. 

Until 2014, only three complete genomic sequences of Seneca Valley virus were available in public databases (GenBank accession numbers NC_011349, DQ641257, KC667560). Therefore, few studies were based on the complete genome of the virus, with the predominant prototype being SVV-001 (GenBank accession number DQ641257) [3,4,5]. These studies led to the definitive classification of Seneca Valley virus as the single representative strain of the species *Senecavirus A* (SVA), genus *Senecavirus* within *Picornaviridae* family [6].

SVV-001 has the typical genome of other picornaviruses, with the standard L-4-3-4, layout, namely Leader—4 polypeptides of the P1—3 polypeptides of P2—and 4 polypeptides of P3. The SVV genomic RNA consists of approximately 7200 nucleotides (nt), with additional 666 nt in the 5′UTR portion and 71 nt in the 3′UTR portion and a poly(A) tail. The virus genome has a single open reading frame (ORF) that encodes a polyprotein of approximately 2180 amino acids (aa) [3]. 

An analysis of the nt sequence within 5′UTR of the SVV-001 prototype revealed high levels of secondary structures [3]. The 5′UTR region of the SVA genome has an internal ribosome entry site (IRES), the function of which is to allow independent translation of the viral RNA by inhibiting the translation of cellular RNA. The 406–625 nt sequence is 57.3% identical to the Hepatitis C virus (*Flaviviridae* family), suggesting that the IRES of senecaviruses is type IV [5]. This finding corroborates previous results, in which several secondary structures from flaviviruses and picornaviruses were identified [7,8,9].

The genomic characteristics of SVA are very similar to the members of *Cardiovirus* genus, especially in relation to the polypeptides P1, 2C, 3C, and 3D. However, SVV-001 differs from cardiovirus in polypeptides 2B and 3A and the IRES type. Genomic regions 2A, 2B, 3A, and 3B of SVV-001 differ considerably from those of all other picornaviruses [3].

Initially, senecaviruses were not associated with any specific pathology. However, many studies have focused on the potential oncolytic activity of senecavirus in human cancer therapy [3,10]. The first evidence that senecaviruses could be associated with porcine vesicular disease was obtained in Canada (2008) and the USA (2012), where symptomatic pigs were detected with the viral RNA by reverse transcription (RT)-PCR assay [11,12]. In both cases, the clinical signs were indistinguishable from vesicular foreign animal diseases, such as foot-and-mouth disease (FMD), swine vesicular disease (SVD), vesicular stomatitis (VS), and vesicular exanthema of swine (VES). These findings prompted the North American animal health authorities to adopt surveillance measures immediately and invest in new studies [13]. 

## 3. Senecavirus Infection in Swine Until 2014

Cases of vesicular diseases of unknown etiologies were reported in pigs in New Zealand [14,15], Australia [16], and the USA [17] in the 1980s. In Europe, reports were performed in the UK in 2007 [18] and Italy in 2010 [19]. In the cases reported in New Zealand and Australia, the occurrence of vesicular lesions in pigs were associated with contact with a green vegetable material infected with the fungus *Sclerotinia sclerotiorum* [14,15] and the feeding of marine products [16]. In the cases that occurred in the USA, the United Kingdom, and Italy, the diagnostic results were negative for the vesicular foreign animal diseases, and the possible cause(s) of the vesicular disease outbreaks in those animals remained unknown. 

Senecavirus was not investigated in any of these cases. In New Zealand, Australia, and the USA, the cases of senecavirus infection were not investigated due to the event dates, since the virus was not known at that time. However, the reasons that the virus was not investigated in the United Kingdom and Italy cannot be ascertained due to the limited data available on the outbreaks in these countries [20]. 

In 2004, outbreaks of vesicular disease were reported in pigs of different production categories in Indiana, USA. Clinical manifestations resembled those of vesicular foreign animal diseases, which were investigated and not detected [21]. Since the etiology of the clinical signs could not be determined, the syndrome was named Porcine Idiopathic Vesicular Disease (PIVD) [21]. 

Pasma, Davidson, and Shaw [11] in 2007 reported that approximately 80% of 187 pigs that were being transported from Canada to the USA presented vesicular lesions that were indistinguishable of those of vesicular foreign animal diseases. FMD, SVD, VS, and VES viruses tested negative in those symptomatic animals. However, senecavirus RNA was detected in the biological samples and was proposed as the etiological agent of the vesicular disease in those pigs [11]. The second detection of senecavirus occurred in 2012 in Indiana, USA, in a single 6-month-old animal with vesicular lesions. This finding reinforced the possible association of senecavirus infection with vesicular disease [12]. 

Although only two reports of senecavirus detection in symptomatic animals were available, a 20-year (1988–2008) retrospective serological study conducted with serum samples of asymptomatic pigs from different states in the USA showed the wide temporal and geographical distribution of senecavirus [3], revealing the silent circulation of the virus throughout the country over the years. 

Considering these studies and the indistinguishable feature of the vesicular lesions associated to senecavirus infection with those caused by the vesicular foreign animal diseases, especially FMD, the United States Animal Health Association elaborated a resolution in which senecaviruses and PIVD are the main subjects, with the aim of developing and implementing plans to minimize the consequences of vesicular lesions not associated with foreign animal diseases found in pigs of that country [13], reinforcing the health and economic importance of these emerging pathogens and disease in swine [20].

## 4. Senecavirus Infection in Swine during/after 2015

**Figure 1:**
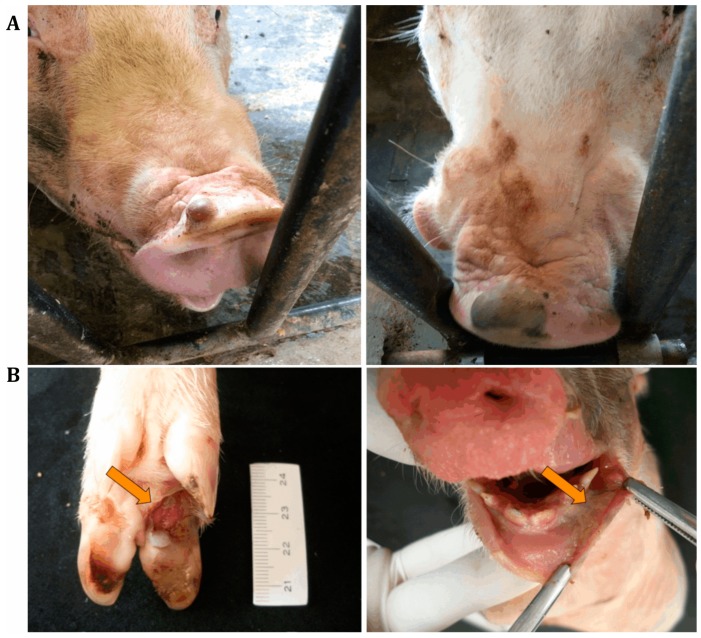
Lesions associated with senecavirus infection. (**A**) Fluid-filled vesicles on the snouts of senecavirus-positive sows. (**B**) Ulcerative lesions on the foot of a three-day-old piglet (**left**) and diphteric gengivitis in a one-day-old piglet (**right**), both positive for senecavirus.

At the end of 2014 and the beginning of 2015, outbreaks of vesicular disease in weaned and adult pigs were reported in different geographical regions of Brazil. Simultaneously, increasing mortality rates of newborn pigs at 1 to 4 days of age were recorded in the main Brazilian pig-producing regions. The reported clinical signs in affected piglets included lethargy, cutaneous hyperemia, diarrhea, neurological signs, and/or sudden death [22,23,24,25]. The Animal Health Department of the Brazilian Ministry of Agriculture, Livestock, and Food Supply (MAPA) provided official tests to screen for foreign animal diseases, specifically FMD, classical swine fever (CSF), and porcine epidemic diarrhea virus (PEDV), but negative results were observed [20,26]. However, other study groups detected senecavirus in pigs with vesicular lesions of different states in the country [20,22,25,27] (Figure 1A), and the virus was considered to be the etiological agent of the reported vesicular disease outbreaks. Additionally, piglets that spontaneously died were submitted to diagnostic investigations and tested negative for different infectious agents that could be associated with one or more of the reported clinical signs, including diarrhea. Since most of the cases were from pig herds with a clinical history of vesicular disease, senecavirus was investigated in piglet biological samples, with positive results being observed in feces, serum, and diverse organ/tissue samples [22,23,24,25] (Figure 1B).

These reports were the first describing senecavirus outside of North American countries. Whether the virus was circulating in Brazil before this period was unknown. However, a 10-year (2007–2016) retrospective serological study conducted in Brazil from serum samples of pigs at different ages suggested that senecavirus was not circulating in the country before 2014, the same year in which senecavirus-associated vesicular disease outbreaks were first reported in the country [28]. 

Senecavirus was first reported outside of the American continent in China in 2015 [29,30]. Pigs in herds located in Guangdong province presented vesicular lesions, and sudden death was observed in newborn piglets. The samples derived from these outbreaks were negative for classic vesicular diseases, but they presented positive results for senecavirus [29,30]. Later, senecavirus infection was reported in symptomatic piglets in other Chinese provinces [31].

**Figure 2:**
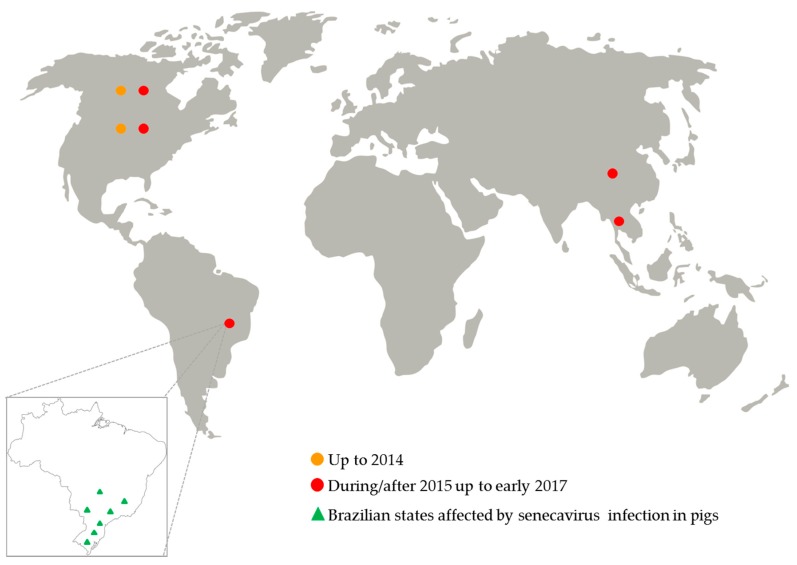
Senecavirus global distribution from 1988 up to early 2017.

Since July 2015, an increased number of senecavirus-vesicular disease outbreaks was reported in the USA [32]; but for the first time in that country, the virus infection was also associated with pig neonatal mortality, especially in pig herds with vesicular disease-affected sows [33,34]. In 2016, pig herds from Canada and Thailand were also affected by senecavirus infection [35,36] (Figure 2).

### 4.1. Epidemiology of Senecavirus Infection

Based on all of these events, 2015 can be considered a turning point for the epidemiology of the senecavirus infection, with many important features of senecaviruses being identified. Until that year, only two cases of senecavirus-induced vesicular disease were reported with a limited number of affected pigs [11,12]. However, during/after 2015 the number of senecavirus infection reports significantly increased and also the morbidity and mortality rates associated with the infection. The morbidity and mortality rates of senecavirus-induced disease vary according to the affected pig category. In a herd that is affected for the first time, the morbidity rates range from 4 to 70% depending on the clinical signs and the pig age groups [20,23,33,34,37]. Senecavirus outbreaks presented morbidity rates of 0.5 to 5% in weaned pigs and 5 to 30% in finishing pigs and breeders [2,20,34], which varied according to the geographical region and the herd origin. Remarkably higher morbidity rates in sows were reported, reaching 70 to 90% [37]. However, the mortality in these categories is very low (≈0.2%), with pigs recovering soon after the remission of clinical signs that last for 10 to 15 days.

In newborn pigs, morbidity and mortality rates are considerably higher, especially in one- to four-day-old piglets, with morbidity rates that can reach 70%, but the mortality rates vary from 15 to 30% [2,23,24,33,34,37]. However, the clinical manifestations and the high mortality rates in piglets last for approximately 2 to 3 weeks in the affected herd. 

To the authors’ knowledge, no re-breaks were reported in previously affected herds, but this is not a permanent condition. The infection likely becomes endemic when most of the animals present asymptomatically and/or with subclinical infection and when clinical manifestations occur in pigs that have not been previously infected, are seronegative, or have low titers of senecavirus-specific antibodies. Furthermore, the declining immunity or the introduction of naive gilts in affected herds and the persistence of the virus in the animal and in the environment may trigger a new outbreak in previously affected herds [2].

After many outbreaks of senecavirus infection were reported in 2015 in the USA, an investigation of 2033 oral fluid samples of asymptomatic pigs from herds located in 25 states of that country revealed that only 1.2% of the samples were RT-PCR-positive for the virus RNA [38]. In contrast, senecavirus RNA was amplified from different biological samples of finishing pigs with vesicular disease. Vesicular fluid and/or the lesions of naturally infected pigs presented high virus loads (2 × 10^7^ to 1.2 × 10^11^ genomic copies/mL) [39], suggesting that direct contact between animals with fluid-filled and/or recently ruptured vesicles and susceptible individuals likely represents one of the most important transmission routes of the virus.

Senecavirus shedding was demonstrated in fecal samples from piglets that presented multisystemic clinical signs, including diarrhea [23,24], and from finishing pigs that were naturally infected with the virus and presented vesicular lesions [39]. Additionally, immunohistochemical and RT-PCR assays revealed the senecavirus presence in the urinary epithelium of clinically affected piglets, suggesting that urine may represent a senecavirus dissemination route and a possible contamination source in affected pig herds [23,24]. 

Senecavirus RNA and viable infective virus particles were isolated in cell cultures from fecal and small intestine samples of mice from clinically affected pig herds in the USA. Houseflies collected from both affected and non-affected herds tested positive for the virus [40]. The detection of the virus genome and/or the isolation of infective senecavirus particles in houseflies and mice suggest that these species may play a role in the epidemiology of the infection [40]. The movement of people, primarily on-farm employee entry, and vehicles, especially those used for the disposal of dead animals and feed delivery, farm tools and equipment, including those used for carcass removal, were also implicated as possible means of introduction and indirect transmission routes of senecaviruses in different pig breeding herds in the USA [37,40]. 

The possibility that senecavirus can be vertically transmitted was raised after the virus was detected by both RT-PCR and immunohistochemical assays from different tissue/organ samples of piglets aged one to two days old [24]. However, further studies are needed to confirm this route of viral transmission. 

**Figure 3:**
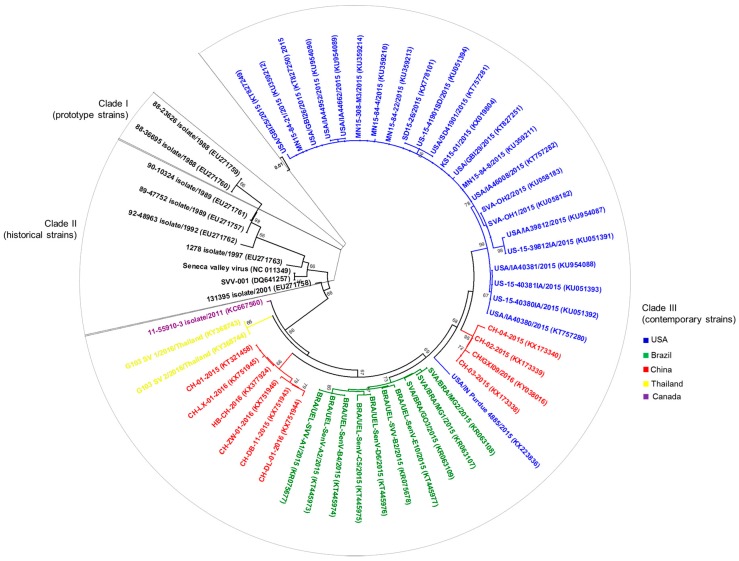
Evolutionary relationships of senecavirus strains identified in the USA, Canada, Brazil, China, and Thailand from 1988 to 2016. Phylogenetic tree constructed with 56 partial (541 bp) nucleotide sequences of the VP1 region of senecavirus genome. Year of sample collection and GenBank accession numbers for each senecavirus strain are presented within the tree. The evolutionary history was inferred using the Neighbor–Joining method [44]. The percentage of replicate trees in which the associated senecavirus strains clustered together in the bootstrap test (1000 replicates) are shown next to the branches [45]. The tree is drawn to scale, with branch lengths in the same units as those of the evolutionary distances used to infer the phylogenetic tree. The evolutionary distances were computed using the Maximum Composite Likelihood method [46] and are in the units of the number of base substitutions per site. Evolutionary analyses were conducted in MEGA7 [47].

After the occurrence and/or the increasing incidence of senecavirus-associated vesicular disease in Brazil, China, the USA, Canada, and Thailand [22,29,30,39,41,42,43] complete (*n* = 42) and partial (*n* = 15) (These numbers were determined based on the complete and partial senecavirus genomic sequences available in GenBank during/after 2015 (Genbank accesses date: 8 June 2017)) genomic sequences were established from senecavirus strains in most of these countries. Therefore, advances were also possible in terms of the molecular epidemiology of the virus. The 42 complete genomic sequences of senecaviruses from 2015 to 2016 showed high nt (95.8 to 99.9%) similarities among each other and lower nt (93.8 to 94.6%) identities with the prototype strain SVV-001. The exception was the Canadian 11-55910-3 strain (GenBank accession number KC667560), which shares 95% and 96–98.2% nt similarities with the prototype SVV-001 and contemporary senecavirus strains, respectively. The grouping of senecavirus strains into three temporal clades was also observed [2,40]. Clade I includes the initially identified senecavirus strains, including SVV-001, clade II includes the USA senecavirus strains identified between 1988 and 1997, and clade III contains the senecavirus strains from Brazil, Canada, China, Thailand, and the USA identified between 2001 and 2016. A phylogenetic tree constructed with all of the currently available nt sequences of the VP1 region of senecaviruses showed a geographical grouping within clade III, with the senecavirus strains clustering according to the country of origin (Figure 3). Despite the divergence between historical and contemporary senecavirus strains, whether genetic changes in the senecavirus genome have led to different biological behaviors of the virus and/or contributed to the emergence of senecavirus infections remains unknown [32,40].

### 4.2. Pathogenesis Evidence of Senecavirus

The definitive association of senecavirus with porcine vesicular disease was shown in experimental infections of 9-week- and 4-month-old pigs with contemporary senecavirus strains [32,48]. In both studies, the incubation period of senecavirus was of 4–5 days [32,48]. The tonsils were indicated as one of the primary sites of senecavirus replication during the acute stage of infection [32]. The viremia period was short, lasting approximately 7 days, and the peak of senecavirus genomic copies (≈1 × 10^6.5^/mL) was detected in serum on the third day post-inoculation (dpi) with a subsequent progressive reduction up to the 10th dpi from when the virus was no longer detected [32]. 

Senecaviruses induce an acute, self-limiting vesicular disease in pigs [32]. Inoculated pigs developed vesicular lesions on the snout, lips, and/or hooves [32,48], specifically on the coronary bands and/or the interdigital space [48]. Other clinical manifestations reported in senecavirus experimental infections include lethargy, lameness, and anorexia [32]. Multifocal deep ulcers and skin erosions/abrasions that evolved to crusted lesions were observed during the infection progress [32,48]. 

Tissue microscopic alterations in experimentally infected pigs were restricted to the tonsils, the spleen, lymph nodes, and the lungs and consisted of mild to moderate lymphoid hyperplasia in the lymphoid tissue and multifocal mild atelectasis and occasionally diffuse congestion with multifocal mild perivascular accumulation of lymphocytes, plasma cells, and macrophages in the lungs [32]. A wide senecavirus tissue distribution was shown, with the presence of virus RNA and infectious particles in the lungs, the mediastinal and mesenteric lymph nodes, the liver, the spleen, the small and large intestines, and the tonsils during the acute phase of the disease. Convalescent pigs were positive for senecavirus RNA in different organs, except for the lungs, the heart, and the liver, but viable virus particles were not isolated from these tissues at a later infection period [32].

According to the experimental studies, senecavirus shedding lasts up to 28 days. The virus can be shed by oral and nasal secretions and feces. The virus excretion peak occurs between 1 and 5 dpi, especially in oral secretions, which presented higher virus loads relative to the nasal secretions and feces [32]. Additionally, senecavirus infectious particles were successfully isolated from oral secretions up to 21 dpi, feces up to 10 dpi, and nasal secretions up to 7 dpi [32]. The combined findings from natural and experimental senecavirus infection suggest that the oral/nasal secretions, feces, and likely urine [23,24] excretions of pigs infected with senecavirus may contribute to the dissemination of the virus both directly, through contact between the animals in the same facility, and indirectly, with the viral agent contaminating the environment. However, further studies are required to confirm the role of these secretions/excretions in senecavirus transmission in field conditions. 

### 4.3. Immunological Response against Senecavirus

Currently there are only a few studies that have evaluated the host immunological response against senecavirus infection. These studies indicate that the senecavirus infection induces early immunological response in clinically affected and non-affected sows under field conditions [34] and in experimentally challenged finishing pigs [32,48]. Seroconvertion to the senecavirus occurs approximately 5 days after the infection, regardless of the clinical manifestations of the disease [32,34,49]. The increasing neutralizing antibody titers occurs simultaneously with decreasing disease severity, viral load in tissues, viremia, and virus shedding, suggesting that antibody responses lead to the progressive clearance of senecavirus from the circulation and most organs, excretions, and secretions [32,34].

The maximum concentration of senecavirus-specific immunoglobulin (Ig) M antibodies lasts for approximately 10 days (from 5 to 15 dpi) followed by a decreasing serological IgM concentration to undetectable titers by 21 dpi [49]. In contrast, the senecavirus-specific IgG response develops later [32,49], with a strong positive response after 21 dpi [49]. 

Different senecavirus antibody titers have been reported in naturally infected pigs and may vary from 160 to 2880 by indirect fluorescent antibody (IFA) or virus neutralization (VN) test [39,50]; but higher titers (≥4096) were detected by the VN test from clinically affected animals in Brazil [28].

### 4.4. Senecavirus Diagnosis

Many diagnostic tests are currently available for the detection of senecavirus infection and/or its association with lesions and disease. Conventional [20,27,40,42] and quantitative RT-PCR (qRT-PCR) [27,33,34,38,39,40,51,52,53] molecular assays are the most used tests in addition to next-generation sequencing (NGS) technology [22,41,51,53]. Molecular assays, especially qRT-PCR, are the gold standards for any vesicular disease, since they are rapid, sensitive, and specific methods that enable the fast and accurate diagnosis of vesicular diseases, including senecavirus genome detection from different biological samples. A disadvantage of senecavirus diagnosis by molecular assay may be the inconsistent detection due to the progression of senecavirus infection, with variations in the viral shedding and/or load in different biological samples [34]. Therefore, a variety of specimens including serum, tonsils, feces, and oral and vesicular fluids, should be collected for senecavirus screening by molecular assays [54].

The other diagnostic tools currently available include immunohistochemical and in situ hybridization, which are assays that identify specific virus antigens and nucleic acid in tissue samples, respectively. Both of these techniques have been successfully used by different study groups for senecavirus-associated disease diagnosis [23,24,25,32,55]. Although histopathological evaluation alone is not conclusive for senecavirus infection diagnosis, findings from this technique may guide the selection of tissue/organ samples for the immunohistochemical and in situ hybridization analyses. 

The antibody detection methods available for senecavirus screening in pigs are indirect [34,49,56] and competitive [49,50] enzyme-linked immunosorbent assay (ELISA), IFA, and VN tests [28,50]. The main advantage of the antibody detection assays is the capability for processing large numbers of samples in epidemiological studies, mass diagnostic programs, and/or in epidemiological surveillance [57], indicating previous virus exposure and/or presence in a herd [56]. 

Regardless of the diagnostic method, senecavirus-associated disease in pigs is clinically indistinguishable from classical viral vesicular infections, especially FMD. The clinical history and/or the presence of lesions that indicate the possibility of vesicular disease should always lead to a complete vesicular foreign animal disease investigation, primarily in the major pig-producing countries, since the emergence of senecavirus infections could adversely affect the prompt reporting of FMD in swine [53].

### 4.5. Prophylaxis and Control Management

Currently, no vaccines or specific treatments are available for senecavirus infections. Therefore, sanitary practices should include prophylactic and control measures that include the herd, the animals, and the environment. Measures should be taken to avoid the introduction of the etiological agent into the pig herds and in those where the infection is established to avoid virus dissemination among the animals of the different pig-producing categories present in the same herd. 

The introduction of senecavirus and other infectious agents can be prevented by adopting strict biosecurity measures. The entry of vehicles, equipment, people, animals, and food into the pig production unit must be strictly controlled [37,40,58]. Livestock trailers and carcass removal equipment were subjectively assessed as the most likely routes of senecavirus introduction in a risk-assessment study [37]. Therefore, the area of vehicle circulation, generally around the farm, should be limited to areas away from the premises where the animals are housed. Preferably, pig transportation should be performed by the same vehicle or livestock trailer, which should not have been in contact with vehicles, personnel, or animals from senecavirus-positive herds [37]. 

Biosecurity measures for people movement events should also be addressed. On-farm employees should shower-in or shower-out of the facility, change clothing and boots prior to entry, and observe a period of downtime after contacting other swine [37]. In the case of animal replacement, pigs should be purchased from farms that are free of the infectious agents of importance in pig health and kept in quarantine before incorporation into the herd. Additional measures include the control of mice and houseflies and the restriction of non-swine domestic animals’ access to the premises within a herd.

In senecavirus-positive farms, in addition to the measures previously mentioned, strict cleaning and disinfection of the facilities and the equipment, an in-pen downtime period, and the all in-all out system have to be adopted. The effectiveness of disinfectants against senecavirus is not yet well-known. Since the clinical signs caused by the senecavirus infection are very similar to those caused by the FMD virus, control measures should be adopted that consider the possibility of FMD virus circulation, including disinfection protocols. This includes the use of sodium hydroxide (2%), sodium carbonate (4%), citric acid (0.2%), acetic acid (2%), sodium hypochlorite (3%), potassium peroxymonosulfate/sodium chloride (1%), and chlorine dioxide [59]. Three disinfectants based on household bleach (Sodium hypochlorite (5.25%)), phenolic derivatives (*p*-tertiary-amilphenol (4%); *o*-benzyl-p-chlorophenol (10%); *o*-phenylphenol (12%)), and quaternary ammonium-aldehyde compounds (alkyl dimethyl benzyl ammonium chloride (26%); Glutaraldehyde (7%)) that have been used against senecavirus were evaluated at different temperatures (4 °C and ≈25 °C) on five different surfaces including cement, rubber, plastic, stainless steel, and aluminum. The results showed that 1:20 diluted household bleach was the most effective disinfectant for the inactivation of the virus at any temperature and on any of the five surfaces evaluated. The disinfectant based on phenolic derivatives was not effective against the virus at any temperature, and the results obtained from the disinfectant based on quaternary ammonia and aldehyde were between those of sodium hypochlorite and phenolic derivatives [60]. 

Accelerated hydrogen peroxide (AHP) is another chemical compound that has been tested as a broad-spectrum disinfectant against bacteria, fungi, and viruses. Disinfectants containing AHP showed virucide activity against some human and animal viruses, such as bovine viral diarrhea virus (BVDV), feline calicivirus, and poliovirus, which also belongs to the Picornaviridae family. In 2016 AHP was shown to be an effective disinfectant against FMD virus, SVD virus, and senecavirus; but its efficacy is dependent on the dilution (1:20) and the contact period (10 min), which are greater than the manufacturer's recommendations [61]. 

Monitoring of the senecavirus circulation should be performed by means of periodical diagnostic examinations conducted on biological samples of symptomatic and asymptomatic pigs from different sites of the production unit. In addition to serological tests, molecular, immunohistochemical, and/or in situ hybridization tests can be performed. Depending on the laboratory methodology to be used, a variety of biological samples such as vesicular and oral fluids and organ/tissue fragments of pigs at different ages, especially tonsils, lymph nodes, spleen, heart, lungs, liver, kidneys, and urinary vesicles, can be analyzed [20,24,32]. The introduction of susceptible pigs in the farm as sentinel animals can also be adopted with great discretion. Suckling animals, especially piglets up to one week of age, should consume adequate quantities of high-quality colostrum and be kept in an appropriate environment that provides comfort and welfare to newborns and sows.

Importantly, the adoption of these measures does not rule out the officially recommended procedures. In 2016, the United States Department of Agriculture (USDA) released a list of recommendations, procedures, and responsibilities for the management of diagnosed or suspected cases of animal vesicular diseases to ensure that investigations into exotic animal diseases occur properly, such that precautions can be taken to prevent the spread of communicable diseases [62]. In Brazil in July 2015, the Department of Animal Health (DSA) of the MAPA published the resolution 018/2015/CGI/DIPOA/SDA with guidelines for procedures to be adopted in the case of clinical signs of vesicular lesions in pigs. The document also guides the veterinarians of the Federal Inspection Departments on how to proceed in the slaughter of pigs that display suspected lesions [63]. However, in order to avoid misinterpretations regarding the conduct to be adopted, the standardization of procedures within and between the pig-producing units is essential [58].

## 5. Conclusions and Perspectives

The studies focusing on the etiological agent and viral infection have considerably expanded the knowledge related to senecavirus and its associated vesicular disease in a short period of time. New study methodologies and tools, particularly the molecular techniques that are notable for the speed, specificity, and sensitivity of their results, have been quickly developed and evaluated. In these studies, some features of senecavirus infection were elucidated in adult animals, and infection in neonates was also described, providing important information on the epidemiology of senecavirus infection, the likely forms of transmission, the pathogenesis, clinical signs, anatomopathological changes, immunity, and a number of suggestions for prophylaxis and control strategies. However, many questions remain to be answered regarding the (i) specific features of the virus, including its biological and molecular evolution, pH stability, receptors and co-receptors, cellular macromolecules, replication cycle, environmental survival; (ii) senecavirus epidemiology, such as how the virus has been disseminated to other countries, non-swine species reservoirs and/or vectors, likely through vertical transmission; (iii) pathogenicity in piglets; and (iv) host immunological response, including the protective antibody titers in colostrum.

Currently, the swine industry, specifically the pig farming sector, is increasingly challenged in terms of animal health and biosecurity. Emerging viral diseases in swine have significantly increased in the past two decades in the global swine population and pose risks to animal health and, consequently, to the production chain [64]. Good examples of economically significant viral agents are porcine reproductive and respiratory syndrome (PRRSV), porcine circovirus type-2 (PCV-2), PEDV, swine influenza virus, and transboundary infectious animal diseases, such as FMD, CSF, and African swine fever [65]. Regarding senecavirus, the available data indicate that the infection is currently limited to the USA, Canada, Brazil, China, and Thailand. The description of the infection in Asia suggests that the senecavirus is not only restricted to a certain geographic region and that senecavirus may well be distributed on a global scale in the future. Therefore, epidemiological investigations should be conducted in countries where the virus has never been reported, especially in those with extensive pig production, where senecavirus infection may have more economic relevance.

Equally important is the capability of the country to detect and diagnose the disease. The rapid establishment of the etiological agent of a disease enables the rapid adoption of control measures, mitigating and/or preventing the local, regional, and/or international dissemination of the infectious agent. Despite the technological advances in the diagnostic field, weaknesses in the veterinary infrastructure of many countries make disease control even more difficult [66].
